# Exploring older people’s end-of-life care preferences over time: A scoping review

**DOI:** 10.1177/02692163251331161

**Published:** 2025-04-25

**Authors:** Lucy Robinson, Felicity Dewhurst, Amy Huggin, Daniel Stow, Charlotte Stenson, Elizabeth Westhead, Katie Frew, Barbara Hanratty, Paul Paes

**Affiliations:** 1 Faculty of Medical Sciences, Newcastle University, Newcastle upon Tyne, UK; 2 Northumbria Healthcare NHS Foundation Trust, Northumberland, UK; 3 St Oswald’s Hospice Newcastle, Newcastle upon Tyne, UK; 4 Wolfson Institute of Population Health, Queen Mary University of London, London, UK; 5 Health Education England North East, Newcastle upon Tyne, UK; 6 Newcastle University, Newcastle upon Tyne, UK

**Keywords:** Advance care planning, aged, patient-centered care, patient preference, review, palliative care

## Abstract

**Background:**

Understanding the evolution of end-of-life preferences over time is important for dynamic, person-centred palliative care. This is particularly relevant for older people whose preferences can be incompletely expressed and subject to change.

**Aim:**

To summarise the nature of the current evidence about how and why the end-of-life preferences of older people change over time.

**Design:**

A scoping review was performed, using a predefined protocol and following the JBI manual for evidence synthesis.

**Data Sources:**

Final searches of Medline, Embase, PsycINFO and Web of Science were carried out in October 2023. Reference lists were also reviewed. Eligibility criteria included studies recruiting people over the age of 60 that explored how or why end-of-life preferences developed over time.

**Results:**

Screening identified 52 articles, reporting on 40 studies. A majority were longitudinal studies collecting quantitative data about treatment preferences. Other preference categories included euthanasia, balancing quality and length of life, goals of care, preferred place of death, decision-making and spiritual preferences. Studies explored a variety of factors that may influence preference change or stability. There was a lack of research with ethnic minority groups and people aged over 80.

**Conclusions:**

Existing research has focused on preferences about specific therapies, at the expense of understanding what matters most to older people. Synthesis of the available evidence about why preferences change will guide reviews of patients’ advance care plans. To inform dynamic, person-centred end-of-life care we need studies prospectively exploring how older people construct a broader range of preferences, and negotiate these over time.

**What is already known about the topic**

- International consensus on advance care planning recommends regular review of individuals’ end-of-life care preferences in physical, psychological, social and spiritual domains.
- Wishes about life-sustaining treatment and place of death change over time for a significant minority but older people’s preferences are complex and may be more subject to change.
- There is a gap in our understanding of how or why older people’s end-of-life care preferences change over time.

**What this paper adds**

- Most research on this topic consists of longitudinal studies quantifying changes in older people’s life-sustaining treatment preferences.
- There is little research outside of Europe or North America and a lack of representation from people aged over 80 or ethnic minority groups.
- A wide variety of factors in physical, psychological, social and spiritual domains have been investigated for their influence on preference change or stability.

**Implications for practice, theory or policy**

- Research is needed on what matters most to older people when they are nearing the end of their lives and how they negotiate this over time.
- Our understanding of the preferences of populations underserved by specialist palliative care is incomplete, including the oldest old and older people from ethnic minority groups.
- Evidence synthesis of the factors influencing whether preferences change or remain stable would inform dynamic advance care planning.

## Background

Patient preferences are an increasingly important influence on clinical practice and healthcare service design. The World Health Organisation emphasises the importance of patients’ individual preferences guiding provision of person-centred palliative care throughout the world.^1^ Preferences refer to patients’ judgements about the desirability of different options or experiences within healthcare.^2^ They can vary from a statement about what a patient would want in a particular circumstance to an expression of more general values.^3^ Preferences can relate to the context in which care is delivered, care relationships, patients’ involvement in their care, or care decisions and care outcomes.^4^ These individual priorities and wishes guide the decisions people make about their health and social care.

One area where preferences are particularly important, is end-of-life care. The term ‘end-of-life care’ is used for care of people who are expected to die within the next year.^5^ This may be within hours or days from an acute illness, or over the next 12 months due to advanced disease, multimorbidity or frailty. End-of-life care can involve decisions that are medically or ethically complex or emotionally challenging, and patients’ preferences are particularly important. Patients’ preferences for end-of-life care are often discussed and recorded as part of advance care planning.^6^ A recent international consensus defined advance care planning as ‘a process which enables individuals to define goals and preferences for future medical treatment and care, to discuss these goals and preferences with family and health-care providers, and to record and review these preferences if appropriate’.^6^ Existing research shows that preferences are not always stable and fixed, especially in situations that are complex and changing.^3^ Therefore, documented wishes may not always reflect currently held preferences and it is important to review advance care planning documents as people’s illnesses progress.^6^ What current evidence does not make clear is how often this review should take place, which aspects it should involve, or which patients should be prioritised.

This is particularly relevant to improving end-of-life care for people dying in later life. This population often has complex or incompletely expressed preferences, which can be more subject to change.^3,4,7^ In addition, older people who are living with frailty often experience a trajectory of prolonged decline with periods of acute illness and uncertainty of prognosis.^8^ This makes recognising when they are approaching the end of their life, and hence when to initiate advance care planning, a challenge. Older people living with frailty may not see themselves as ill enough to plan for future healthcare, or see it as futile.^9^ Therefore, these concepts need to be introduced gradually and in a way that is responsive to changing circumstances. Findings from research into preferences in one group of patients may not be transferable to a group with different diagnoses and therefore different illness experiences. For example, older patients and people with non-malignant conditions are less likely to prefer home death than those with cancer.^7^ A recent systematic review, of the factors influencing the formation of older people’s care preferences, suggests that the influences on preference change or stability over time remain unclear.^4^ Understanding how and why preferences change or remain stable in this population will help to provide advance care planning which is relevant, up to date and more responsive to older people’s changing needs.

Existing systematic reviews about longitudinal changes in end-of-life preferences have focussed on specific, isolated topics, for example preferred place of death^10^ or treatment preferences.^11^ However, preferences about care in the last year of life are broad and can relate to many different domains within healthcare.^3^ We found no systematically constructed reviews that have summarised the various ways in which research has explored change or stability of end-of-life care preferences, and none focussing on longitudinal preferences in an older population. This scoping review aims to provide an overview of this important topic, to identify key concepts and guide areas for future research.^12^

### Review question

‘What is the nature of the current evidence about how and why the end-of-life care preferences of older people change over time?’

### Objectives

1. To systematically identify research that explores:
  a. How the end-of-life care preferences of older people change over time
  b. What influences any change or stability in their preferences
2. To summarise the key ways in which this research explores the concept of end-of-life care preferences over time
3. To identify gaps in the current literature on this topic, to aid planning of future systematic reviews or primary research in this area

## Methods

We conducted this scoping review according to a predefined protocol, published on Open Science Framework in January 2022 (https://osf.io/9mb5f). There were no deviations from this protocol. We followed the JBI Manual for Evidence Synthesis^13^ and the Preferred Reporting Items for Systematic reviews and Meta-Analyses extension for Scoping Reviews (PRISMA-ScR) Checklist^14^ (Supplemental Table 1).

### Literature search

We developed the search strategy in collaboration with a research librarian and experts in ageing research and palliative medicine. The strategy combined Medical Subject Headings (MeSH) and keywords related to older people, end-of-life care and preferences. We used search strategies from related reviews to inform the search terms. The initial search strategy was trialled in Medline and we used terms found in the key papers identified to refine the search strategy. We reviewed the final search strategy with the Peer Review of Electronic Search Strategies (PRESS) checklist.^15^ The full search strategy can be found in Supplemental Table 2. There were no search restrictions on date, language or type of research study. This search strategy was adapted for each database and we performed the final searches in Medline, Embase, PsycINFO and Web of Science in October 2023.

Following study selection, we examined the reference lists of articles included in the review for additional references. We contacted authors of included conference abstracts, if details required for the review had not been subsequently published.

### Evidence screening and selection

We selected evidence based on the pre-specified inclusion and exclusion criteria described in Table 1. We included studies which either explored how preferences change over time or whether particular factors impact on preference change or stability. We defined older people as those aged 60 or over, because it is the age threshold used by the World Health Organisation.^16^ However, we recognised that a chronological age threshold to define older people is something that can differ between countries or change over time.^17^ Therefore, we maintained flexibility in our approach and included studies that stated they were researching an older population but used a younger chronological age threshold. We defined end-of-life care preferences as any preference about care in the last year of someone’s life.^5^ This was because we wanted to present a broad overview of how end-of-life preferences are conceptualised in the literature. We excluded any studies which took preference measurements before and after an experimental preference intervention, for example a new advance care planning initiative.

**Table 1.** Inclusion and exclusion criteria.

Inclusion criteria 1. Studies with people aged 60 years or older. Mixed age studies (where data from people aged ⩾60 were analysed separately) were included. Articles which stated they were researching an older population, but with a younger chronological age threshold, were included. 2. Studies investigating how older people’s end-of-life care or treatment preferences change over time. OR 3. Studies which explore why older people’s end-of-life care or treatment preferences change or remain stable. 4. Original research studies or systematic reviews of original research. Exclusion criteria 1. Studies which include participants under the age of 60, unless their data are analysed separately. 2. Studies which are investigating the preferences of relatives or health care professionals. 3. Studies which focus on general care or treatment preferences, rather than those in the last year of life. 4. Studies which explore care and treatment preferences at one specific point in time, and do not investigate how or why preferences change or remain stable. 5. Studies which are validating a tool or investigating the effect of an intervention on participants’ preferences. 6. Studies exploring the process of decision making, rather than what people’s preferences are and how they may or may not change over time. 7. Descriptive case studies, editorials, book chapters, opinion pieces or guidelines (relevant original case study research will be included).

Search results were imported into Endnote, and duplicates removed.^18^ Using Rayyan software, one reviewer (LR) screened all titles and abstracts for inclusion and a second reviewer (AH, BH, KF or PP) independently screened 10% of the titles and abstracts. Any disagreements were resolved by discussion with a third reviewer. There was a 98% agreement rate at this stage and discussion further clarified the application of the inclusion and exclusion criteria to the literature.

All the full texts of references included at the title and abstract screening stage were screened for eligibility by two reviewers (LR, FD, AH, DS, CS, EW, KF, BH or PP). Any disagreements were resolved by discussion with a third reviewer.

### Data charting and analysis

Two reviewers (LR and FD) piloted the data charting table at the protocol development stage. Due to the iterative nature of scoping reviews, we refined the data charting and analysis process as the review was conducted and our knowledge of the literature improved.^12^ Following pilot testing, we added extra tables to the data charting template to record details of treatment preferences, hypothetical scenarios and any factors investigated as correlates for preference change or stability. During charting, we found variability in the reporting of study population age, as some studies reported age at baseline and others at follow up. Therefore, we added a category of ‘recruitment age threshold’ to the data charting template. We also added the categories of participant gender and participant ethnicity to provide more information about study populations. The final data charting template can be found in Supplemental Table 3.

One reviewer (LR) extracted the data and this was checked and verified by a second reviewer (FD, AH, DS, CS, EW, KF, BH or PP). Any discrepancies were resolved by discussion. To summarise the key ways in which changing preferences have been explored in this population, data charting focussed on study methodology and key concepts, such as how preferences were defined.

We used descriptive statistics to summarise the characteristics of the studies included in the review and descriptive content analysis to categorise the key concepts identified in this area of research.^19^ When coding for types of preference measured by the studies, we considered existing literature identifying preference categories^3,20^ but also kept an open mind about new categories emerging from our data. We presented the results in tabular format and diagrammatically.

## Results

**Figure 1.**
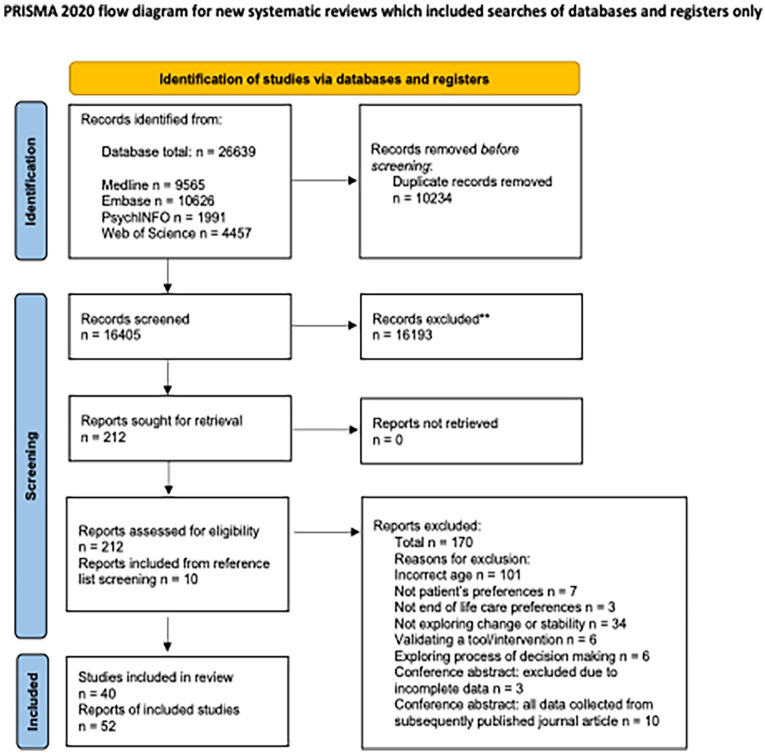
PRISMA flow chart.^21^

The PRISMA flow diagram (Figure 1) shows the results of the searches and screening process. Following deduplication, 16,405 references were screened using titles and abstracts. Two reviewers then independently screened 212 full texts and 45 references were included in the review. Reference list screening of these articles revealed 10 further references for inclusion, bringing the total to 55. Three of the 55 references were conference abstracts without subsequently published results and, as we had no response after contacting the authors, we excluded all three due to incomplete data. This left 52 references for inclusion, which reported on 40 studies.

The included articles were published between 1994 and 2023. The key characteristics and results of the 52 included references are summarised in Supplemental Table 4. The 52 references reported on 40 studies, comprising 39 primary research studies and one related systematic review. This systematic review synthesised the literature about the stability of end-of-life treatment preferences in all adult patients, but included a subgroup analysis of three studies with older adults.^11^ As these three studies were included in our review in their own right we excluded this systematic review from further analysis. The following sections describe the results of our analysis of the 39 primary research studies.

### Type of evidence

Table 2 summarises the characteristics of the 39 studies included in further analysis. Nineteen studies were conducted in North America, fifteen in Europe, three in Asia and two in Australasia. Nineteen studies recruited participants from Primary Care, eighteen from Secondary Care and two from Primary and Secondary Care. Primary Care was defined as services providing community care, which are usually a patient’s first point of contact^22^ Secondary Care was defined as services providing specialist or emergency care, usually in a hospital setting, and mental health services.^22^

**Table 2.** Summary of characteristics of included studies.

Characteristic and related categories	Number	%
Recruitment context (*n* = 39 studies)		
Primary care	19	48.7
Secondary care	18	46.2
Primary and secondary care	2	5.1
Study design (*n* = 39 studies)		
Quantitative		
Longitudinal (observational)	26	66.7
Interventional	1	2.6
Qualitative		
Longitudinal (observational)	3	7.7
Cross-sectional (observational)	5	12.8
Mixed method		
Longitudinal (observational)	4	10.3
Prospective or retrospective (*n* = 39 studies)		
Prospective	29	74.4
Retrospective	6	15.4
Both	4	10.3
Method of data collection (*n* = 45, 6 studies used 2 methods of data collection)		
Structured interview	22	48.9
Semi-structured interview	10	22.2
Structured questionnaire	8	17.8
Medical record review	2	4.4
Semi-structured focus group	1	2.2
Phenomenological interview	1	2.2
Textual data collection	1	2.2

The most frequently occurring study design was a longitudinal observational study collecting quantitative data (66.7% of studies) followed by a cross-sectional qualitative study (12.8% of studies). Thirty-eight out of the 39 studies were observational. The one interventional study collected longitudinal quantitative data about patients’ preferences during a randomised controlled trial comparing standard and intensive medical therapy in heart failure.^23^ Preference data was collected in both arms of the study and analysed without reference to the medical intervention and therefore this study was included in the review.

A majority of studies (34/39) were longitudinal. The five cross-sectional studies were qualitative studies exploring retrospectively how or why participants’ preferences had evolved over time. There were only three qualitative longitudinal studies; one exploring the reasons for preference change or stability using a preference based card game,^24^ the second exploring how spiritual needs and preferences change over time^25^ and the third exploring participants’ experiences of having a ‘wish to die’ and how this evolved over time.^26^ Two of the longitudinal mixed methods studies collected data about treatment preferences^27,28^ and two collected data on pre-defined patient goals at each assessment and then qualitatively explored why these goals changed or stayed the same over time.^29,30^

### Participant characteristics

Twenty-seven (69%) studies recruited participants living in the community, four of these specifically recruited nursing home residents. Eight studies (21%) recruited hospital inpatients, seven of these recruited acute hospital inpatients and one study recruited psychiatric hospital inpatients. Four studies (10%) recruited both hospital inpatients and community dwelling outpatients. Two of these studies recruited medical patients and two studies recruited psychiatry patients.

Regarding the health status of participants, 18 studies (46%) recruited older adults living in the community and their recruitment was not based on their medical diagnoses. Ten studies (26%) recruited older adults with particular medical diagnoses. These included heart failure, chronic obstructive pulmonary disease, cancer, multimorbidity and frailty. Seven studies (18%) recruited patients admitted to an acute medical hospital and three studies (8%) recruited patients with a psychiatric diagnosis. One study (3%) recruited patients enrolled in a community hospice programme.

In the 38 studies which provided information on participant gender, an average of 53.8% of the participants were female. Twenty studies recorded the race or ethnicity of participants and in these studies 89% of participants identified as white ethnicity.

### Definition of ‘older people’

**Figure 2.**
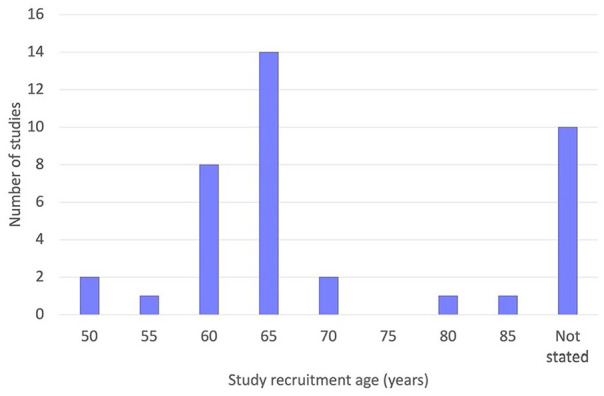
Recruitment age thresholds used by the studies included in the review (*n* = 39).

Twenty-nine of the 39 studies recruited participants over a specified age. The distribution of age thresholds is shown in Figure 2. The most common recruitment age was 65 or over (14/39 studies). The remaining 10 studies did not state a recruitment age but included participants from a particular group of older adults, for example, geriatric inpatients.

### How preferences change: Preference categories

All of the included studies assessed whether certain end-of-life preferences changed or remained stable. Thirty-one studies observed preferences over time, eight studies measured preferences before and after an event, and four studies retrospectively explored how preferences had changed at the very end of people’s lives. Four studies employed two of these methods.

There were 48 types of preference measurement across the 39 studies. Six studies measured more than one type of preference. Twenty-six (54%) preference measurements related to treatment preferences; five (10%) to preferences for physician assisted suicide, euthanasia or having a ‘wish to die’; and five (10%) asked participants to make judgements about whether they would prioritise quality or length of life in certain circumstances. Four (8%) preference measurements asked participants about their preferred place of death, four (8%) about general end-of-life care goals, three (6%) discussed decision-making preferences and one (2%) explored how spiritual preferences change over time.

**Figure 3.**
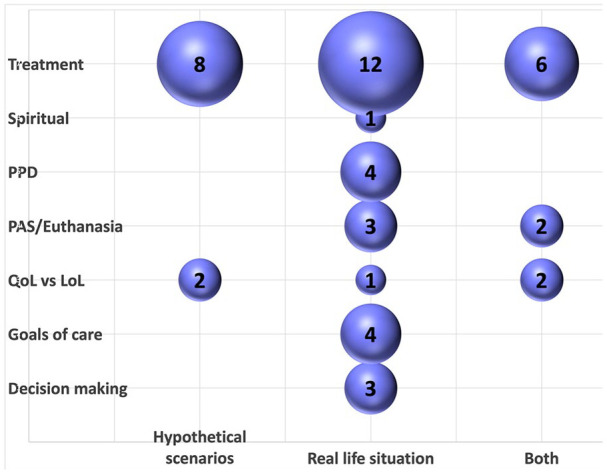
Content analysis of preference measurements. This bubble plot shows the results of the content analysis of the 48 types of preference measurement made across the 39 included studies. It plots each preference measurement according to its preference category and whether the preference was measured in real or hypothetical circumstances. The size of the bubble represents the number of preference measurements in each category. PPD: preferred place of death; QoL: quality of life; LoL: length of life; PAS: physician assisted suicide.

Figure 3 displays the 48 types of preference measurement and whether they were made for participants’ real-life circumstances or hypothetical scenarios. The majority of preference measurements were made considering participants’ real-life situation.

### Why preferences change: Factors influencing preference change or stability

**Figure 4.**
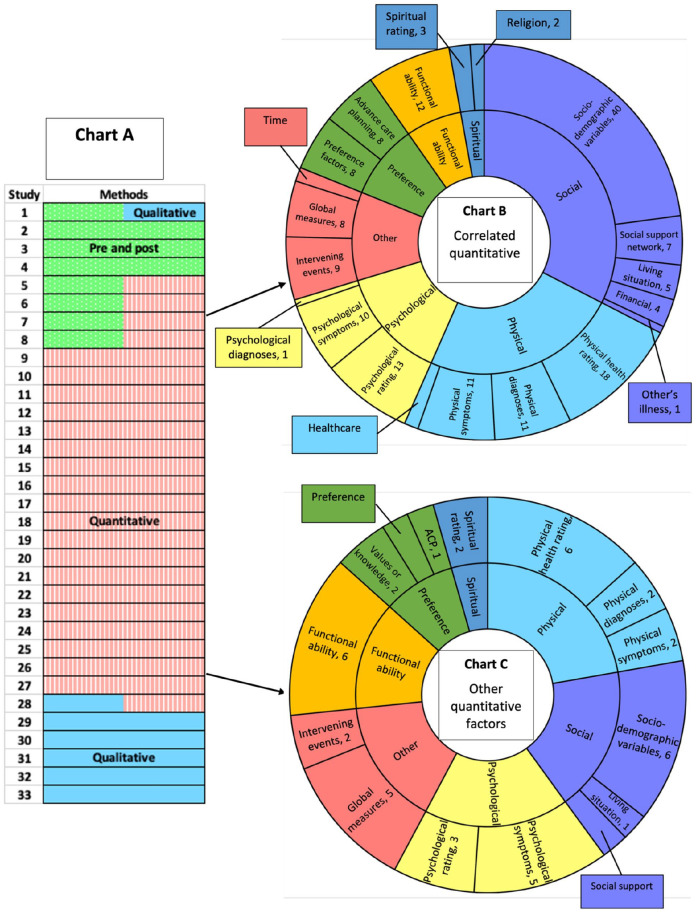
Analysis of why preferences change or remain stable. ACP = advance care planning. Chart A (Methods) shows the different methods used by the 33 studies which investigated why preferences change or remain stable. Green with dots = Measured preferences pre and post a change in circumstances, red with lines = measured quantitative factors that may influence preference change or stability, blue solid = collected qualitative data about why preferences change. Six studies used more than one method. Chart B (Correlated quantitative factors) shows the content analysis of the factors that were statistically analysed for correlation with preference change or stability. *N* = number of times a factor in this category was analysed across the 15 studies that used this type of analysis. Chart C (Other quantitative factors) shows the content analysis of the quantitative factors which were compared with preference change or stability but not analysed for statistical correlation. *N* = number of times a factor in this category was used across the 13 studies that collected this type of data.

Thirty-three studies investigated potential reasons for preferences changing or remaining stable over time. The methods used were measuring preferences pre and post a change in circumstances, collection of qualitative data, collection of quantitative data about factors which may influence preferences and statistical analysis of these factors for correlation with preference change or stability (Figure 4, Chart A).

The eight studies which measured preferences before and after a change in circumstances investigated the following as potential precipitants for change: treatment of depression (4 studies), hospice enrolment (2 studies), a hospital admission (1 study) and introduction of financial constraints (1 study). All eight studies assessed how preferences had changed following these events. Four of the studies used quantitative analysis to investigate additional factors that may be associated with preference change or stability. One study also collected qualitative data on why preferences changed (Figure 4, Chart A).

Seven studies inductively explored why preferences changed or remained stable by collecting qualitative data. Most of these studies (57%) were exploring general goals of care.

Twenty-four studies presented quantitative data on why preferences change or remain stable. The majority of these studies (75%) were investigating stability of treatment preferences. Studies performed different levels of quantitative analysis on this data. Some studies calculated the preference stability in different groups of participants, others collected quantitative data on factors which may influence whether preferences change or remain stable. Fifteen of the twenty-four studies went on to statistically analyse the correlation between certain factors and preference change or stability. Examples of these factors include age, quality of life scores and functional status. We performed content analysis on all the factors that were quantitatively compared with preference change or stability. We differentiated between factors which were measured alongside preference change and those which were statistically analysed for correlation with preference change or stability. Any factor analysed as a potential correlate of change or stability of preferences is shown in Figure 4, Chart B. Factors that were measured in relation to preference change but not statistically analysed for correlation are shown in Figure 4, Chart C. The most commonly investigated factors were sociodemographic variables and physical health ratings. However, a broad range of factors were studied in physical, psychological, social and spiritual domains. Several studies considered ‘preference’ factors, for example whether the strength or type of the participant’s original preference influenced preference change, or whether writing preferences down as part of advance care planning impacted preference stability. Studies also considered functional ability and ‘other’ factors, such as global quality of life scores, follow up time and events occurring between interviews.

## Discussion

### Main findings

This review summarises the current literature exploring change or stability in older people’s preferences for care in the last year of their life. A majority of studies were of longitudinal, prospective, observational design, collecting quantitative data. Most of the research was conducted with community dwelling, white older adults in North America or Europe. A threshold age of 60 or 65 was commonly used to define ‘older’ people.

Regarding the type of preferences investigated, the majority of studies assessed whether specific treatment preferences change or remain stable over time. There were four studies assessing more general end-of-life goals of care. All but one asked participants to rate pre-defined care preferences and then explored why they either changed or remained stable. Other topics included how preferences for euthanasia change over time, weighing up quality or length of life and preferred place of death. There were only three studies exploring decision-making preferences and one study about spiritual preferences.

The included studies investigated a significant number of factors which may influence preference change or stability. These included intervening events, factors in physical, psychological, social and spiritual domains, and advance care planning.

### What this study adds

#### Suggested targets for future evidence synthesis

Despite the recent international consensus definition of advance care planning advocating for a broader approach,^6^ the majority of research in this area consists of quantitative studies analysing how older people’s preferences for life-sustaining treatments change over time. Systematic review and meta-analysis of these results may help to clarify the temporal validity of advance care planning documents that state older people’s specific treatment preferences. These documents, examples of which include Living Wills and Advance Decisions to Refuse Treatment, enable patients to document their wishes about common medical treatments, in the event that they lose capacity to make decisions for themselves.^31,32^ They are legally binding in some circumstances. Synthesis of the data from existing studies could clarify how likely it is that older people’s documented treatment preferences will change, but there are several issues with translating this into individual care. Firstly, there is evidence that choices about specific treatments do not always reflect older patients’ underlying values.^33^ This calls into question the real-life validity of the specific treatment preferences measured by these studies. Secondly, research shows that it can be difficult to make clear advance decisions for treatment due to problems predicting our response to future circumstances.^34^ Patients may adapt to illness as it progresses, making it difficult to accurately predict how they will feel when experiencing a particular health state.^35^ These challenges may be exacerbated for older people living with frailty, who often experience a more uncertain illness trajectory.^8^ Thirdly, by focussing on treatment preferences this meta-analysis would not reflect the broad and varied nature of preferences for care in the last year of life.

Most studies measured preferences in participants’ real-life situation, which address these validity concerns to some extent. However, many of the studies were community based cohort studies, where participants were not facing the complexities of end-of-life decision making. Recruiting research participants who are unwell is challenging, as patients may have complex symptoms or an unstable illness course.^36^ However, research has shown that people who are dying identify several benefits to taking part in end-of-life research^37^ and appropriate methodologies continue to develop.^38^ This debate reflects the important dilemma of when it is best to complete advance care planning, as people who are dying may be too unwell to express their preferences and therefore it is important to understand their wishes in advance, but preferences elicited too early may change.^39^ These complexities highlight the importance of the tailored approach to advance care planning advocated by recent international consensus, where conversations about end-of-life care are introduced at a time appropriate to each individual and repeated to regularly review preferences.^6^ However, when to revisit these conversations within a resource limited healthcare system remains unclear. Our review identified 33 studies that explored why preferences change. A mixed method systematic review synthesising the results of these studies would inform this tailored approach. Instead of quantifying preference stability in a particular population, it would seek to identify triggers for preference change, guiding healthcare professionals on when they should revisit advance decisions with older patients. Across the 33 studies there is significant heterogeneity in the preferences, scenarios and populations studied but a mixed method evidence synthesis would allow us to explore the impact of these differences on the results. This synthesis could build upon the work of Etkind et al.,^29^ who produced a model of influences on preference stability for older adults with frailty using a longitudinal mixed method cohort study.

#### Areas for future research

This scoping review reveals several areas for future research including studies recruiting populations currently underserved by specialist palliative care, decision-making preferences and understanding what matters most to older people at end of life, including preferences in psychological, spiritual and social domains.

Despite having no restrictions on language, we found little research from Africa, Asia, Australasia and South America. In part, this may be a result of cultural differences in end-of-life care decision making, with less emphasis placed on individual patient’s preferences in some healthcare contexts.^40^ This review focussed on patient preference and decision making, which meant that studies investigating surrogate decision making were out of scope. This may explain why the majority of included studies were from Europe and North America, where patient autonomy is highly valued within medical care.^41^ In other cultures it is more common for family members to have a strong influence on, and in some cases responsibility for, end-of-life care decision making.^40,42^ Performing a complementary scoping review, to explore how families’ wishes for their older relatives’ end-of-life care change over time, would inform family-centred palliative care worldwide.^43,44^

Even in cultures where personal autonomy is highly valued, older people’s individual preferences for control over decision making are important to consider. Healthcare decision making is recognised as a spectrum from full individual autonomy through supported decision making to surrogate or substitute decision making, where someone else makes the decision on a patient’s behalf.^45^ A recent review summarising the end-of-life care preferences of older people with multimorbidity included several cross-sectional studies investigating whether patients wished to be involved in shared decision making.^46^ Our review highlights the lack of longitudinal research in this area. This is a topic that warrants further investigation. Studies have shown that older patients are more likely than younger patients to want healthcare professionals or family members to make decisions on their behalf,^47–49^ but whether older people’s preferences for decision making change as their illnesses progress remains unclear. This is particularly relevant to end-of-life care, because as people become more unwell they are more likely to develop conditions that impact on their decision-making capacity.^50^ It will be important for future research to acknowledge the continuum of decision making and the common need for support with decisions in the last year of life, rather than considering decision making a dichotomy between autonomy and surrogate decision making.^45^

The lack of research conducted outside of a North American or European setting may also reflect the global inequality of palliative care provision, and therefore research. The World Health Organisation estimates that only 14% of people who need palliative care globally receive it.^1^ Preferences require people to have a choice about their healthcare, but robust healthcare systems providing choice should also be based on what is important to the patients they serve. It is important that palliative care capacity building in low and middle income countries includes research into individuals’ needs and preferences and how these change over time.^51^

Inequalities in palliative care access also persist in resource rich countries.^52^ Populations currently underserved by specialist palliative care include people with a non-cancer diagnosis, people from ethnic minority or LGBTQI+ communities, those incarcerated or homeless and the ‘oldest old’.^52–54^ The studies included in this review recruited participants with a range of diagnoses, expanding the evidence base beyond patients with a diagnosis of cancer. However, few participants were from ethnic minority backgrounds and there was no record of research with older LGBTQI+, homeless or prisoner communities, which needs to be addressed.

As this review focuses on older people, it is important to consider the ‘oldest old’, a term used to describe people over the age of 80, or over the age of 85 in some countries.^55,56^ Only two of the included studies specifically recruited participants over the age of 80. Globally, the number of people over 80 is predicted to triple from 143 million in 2019 to 426 million in 2050.^57^ People of this age are more commonly living with frailty and multimorbidity^58^ and may have different priorities than the ‘younger old’, who are more likely to still be working or caring for grandchildren or parents.^59,60^ Research based on the ‘younger old’ may not translate to the ‘oldest old’, therefore studying the unique experiences that influence change or stability of preferences in this age group is essential.^61^

In addition to calling for a regular review of preferences, the recent consensus definition recommends expanding advance care planning beyond eliciting specific treatment preferences.^6^ It emphasises planning that discusses individuals’ values and how these impact goals and preferences for future care in physical, psychological, social and spiritual domains.^6^ The small number of studies investigating preferred place of death, goals of care and spiritual preferences reveals a need for evidence about how older people negotiate preferences outside the physical domain. There is a dearth of longitudinal qualitative studies investigating how older people themselves view what matters most in the last year of their lives. The validity of data collected by cross-sectional qualitative studies may be impacted by false memories of previous preferences, which are surprisingly common.^62^ Studies which use previous literature or patient and public involvement to define care goals, and then measure how and why these change over time add valuable insight.^29,30^ However, given that older people’s preferences can be incompletely expressed and emerge from a complex interaction of factors,^4^ inductive exploration of how these wishes form and evolve over time would add rich understanding to this area of research. Qualitative studies prospectively exploring how older people construct a broader range of preferences and negotiate these over time will inform dynamic and person-centred advance care planning.

### Strengths and limitations

This scoping review used a sensitive search strategy with broad search terms to identify the many different ways in which preferences are conceptualised in the literature. This meant that the number of references retrieved by the searches precluded double reviewer screening of all titles and abstracts due to resource constraints. However, there was a high level of agreement in the 10% of abstracts that were double screened and this process clarified the application of the inclusion and exclusion criteria. The broad and varied search terms used for preferences means we have provided a comprehensive overview of how research has explored this phenomenon.

This review used an age threshold of 60 to define older people. We also included studies that stated they were researching an older population but used a younger chronological age threshold. This was a strength because it enabled us to provide a global view of how older people are defined in the literature; this definition may change from country to country depending on average life expectancy.^17^ A potential limitation is that because we used an age threshold rather than average age, we excluded papers where it was not clear that all participants were over a particular age. This has the potential to exclude research papers where the majority of participants were older people, if the authors only presented an average age rather than a range or recruitment age threshold for their study. However, this decision means that we have provided a robust review of the studies aiming to research an older population specifically. As we have discussed in the areas for future research section of this paper, this review’s focus on patient preference and personal autonomy may be less applicable in some healthcare contexts. Therefore, a complementary review investigating how the preferences of surrogate decision makers change over time is recommended.

In conclusion, this review provides a comprehensive overview of the different ways in which the current literature has explored how the end-of-life preferences of older people evolve over time. It identifies several areas where future research could build upon our current understanding including: evidence synthesis of factors influencing preference change or stability, research in older populations currently underserved by specialist palliative care and a need for inductive longitudinal studies collecting qualitative data. Expanding the evidence base in this way will inform authentic, dynamic and person-centred end-of-life care for this fast growing section of the global population.

## Supplemental Material

sj-docx-1-pmj-10.1177_02692163251331161 – Supplemental material for Exploring older people’s end-of-life care preferences over time: A scoping review Supplemental material, sj-docx-1-pmj-10.1177_02692163251331161 for Exploring older people’s end-of-life care preferences over time: A scoping review by Lucy Robinson, Felicity Dewhurst, Amy Huggin, Daniel Stow, Charlotte Stenson, Elizabeth Westhead, Katie Frew, Barbara Hanratty and Paul Paes in Palliative Medicine
