# Supplementary material for: Exploring older people’s end-of-life care preferences over time: A scoping review
Source: Palliat Med. 2025 Apr 25;39(6):665–77. doi: 10.1177/02692163251331161 (PMC12102515; doi:10.1177/02692163251331161)
Supplement: sj-docx-1-pmj-10.1177_02692163251331161 – Supplemental material for Exploring older people’s end-of-life care preferences over time: A scoping review [file sj-docx-1-pmj-10.1177_02692163251331161.docx]

Supplementary Materials

Supplementary Material Table 1: PRISMA-ScR Checklist

**Preferred Reporting Items for Systematic reviews and Meta-Analyses extension for Scoping Reviews (PRISMA-ScR) Checklist**

| **SECTION** | **ITEM** | **PRISMA-ScR CHECKLIST ITEM** | **REPORTED ON PAGE #** |
| --- | --- | --- | --- |
| **TITLE** | | | |
| Title | 1 | Identify the report as a scoping review. | 1 |
| **ABSTRACT** | | | |
| Structured summary | 2 | Provide a structured summary that includes (as applicable): background, objectives, eligibility criteria, sources of evidence, charting methods, results, and conclusions that relate to the review questions and objectives. | 2 |
| **INTRODUCTION** | | | |
| Rationale | 3 | Describe the rationale for the review in the context of what is already known. Explain why the review questions/objectives lend themselves to a scoping review approach. | 3-4 |
| Objectives | 4 | Provide an explicit statement of the questions and objectives being addressed with reference to their key elements (e.g., population or participants, concepts, and context) or other relevant key elements used to conceptualize the review questions and/or objectives. | 4 |
| **METHODS** | | | |
| Protocol and registration | 5 | Indicate whether a review protocol exists; state if and where it can be accessed (e.g., a Web address); and if available, provide registration information, including the registration number. | 4 |
| Eligibility criteria | 6 | Specify characteristics of the sources of evidence used as eligibility criteria (e.g., years considered, language, and publication status), and provide a rationale. | 5 |
| Information sources* | 7 | Describe all information sources in the search (e.g., databases with dates of coverage and contact with authors to identify additional sources), as well as the date the most recent search was executed. | 4 |
| Search | 8 | Present the full electronic search strategy for at least 1 database, including any limits used, such that it could be repeated. | 19-21 |
| Selection of sources of evidence† | 9 | State the process for selecting sources of evidence (i.e., screening and eligibility) included in the scoping review. | 5 |
| Data charting process‡ | 10 | Describe the methods of charting data from the included sources of evidence (e.g., calibrated forms or forms that have been tested by the team before their use, and whether data charting was done independently or in duplicate) and any processes for obtaining and confirming data from investigators. | 6 & 22 |
| Data items | 11 | List and define all variables for which data were sought and any assumptions and simplifications made. | 6 & 22 |
| Critical appraisal of individual sources of evidence§ | 12 | If done, provide a rationale for conducting a critical appraisal of included sources of evidence; describe the methods used and how this information was used in any data synthesis (if appropriate). | N/A |
| Synthesis of results | 13 | Describe the methods of handling and summarizing the data that were charted. | 6 |
| **RESULTS** | | | |
| Selection of sources of evidence | 14 | Give numbers of sources of evidence screened, assessed for eligibility, and included in the review, with reasons for exclusions at each stage, ideally using a flow diagram. | 7 |
| Characteristics of sources of evidence | 15 | For each source of evidence, present characteristics for which data were charted and provide the citations. | 23-31 |
| Critical appraisal within sources of evidence | 16 | If done, present data on critical appraisal of included sources of evidence (see item 12). | N/A |
| Results of individual sources of evidence | 17 | For each included source of evidence, present the relevant data that were charted that relate to the review questions and objectives. | 23-31 |
| Synthesis of results | 18 | Summarize and/or present the charting results as they relate to the review questions and objectives. | 7-12 |
| **DISCUSSION** | | | |
| Summary of evidence | 19 | Summarize the main results (including an overview of concepts, themes, and types of evidence available), link to the review questions and objectives, and consider the relevance to key groups. | 13-15 |
| Limitations | 20 | Discuss the limitations of the scoping review process. | 15 |
| Conclusions | 21 | Provide a general interpretation of the results with respect to the review questions and objectives, as well as potential implications and/or next steps. | 15 |
| **FUNDING** | | | |
| Funding | 22 | Describe sources of funding for the included sources of evidence, as well as sources of funding for the scoping review. Describe the role of the funders of the scoping review. | 15 & 23-31 |

JBI = Joanna Briggs Institute; PRISMA-ScR = Preferred Reporting Items for Systematic reviews and Meta-Analyses extension for Scoping Reviews.

* Where *sources of evidence* (see second footnote) are compiled from, such as bibliographic databases, social media platforms, and Web sites.

† A more inclusive/heterogeneous term used to account for the different types of evidence or data sources (e.g., quantitative and/or qualitative research, expert opinion, and policy documents) that may be eligible in a scoping review as opposed to only studies. This is not to be confused with *information sources* (see first footnote).

‡ The frameworks by Arksey and O’Malley (6) and Levac and colleagues (7) and the JBI guidance (4, 5) refer to the process of data extraction in a scoping review as data charting*.*

§ The process of systematically examining research evidence to assess its validity, results, and relevance before using it to inform a decision. This term is used for items 12 and 19 instead of "risk of bias" (which is more applicable to systematic reviews of interventions) to include and acknowledge the various sources of evidence that may be used in a scoping review (e.g., quantitative and/or qualitative research, expert opinion, and policy document).

*From:* Tricco AC, Lillie E, Zarin W, O'Brien KK, Colquhoun H, Levac D, et al. PRISMA Extension for Scoping Reviews (PRISMAScR): Checklist and Explanation. Ann Intern Med. 2018;169:467–473. doi: 10.7326/M18-0850.

Supplementary Material Table 2: Full search strategy

Medline

**MeSH headings exp= Explode** Truncation*

|  | Category and definition | Search terms (Medline) |
| --- | --- | --- |
| 1 | Older people | **Aged exp (includes Aged, 80 and over &**  **Frail elderly)**  Elder*  Older, Oldest Old, Very Old  Seniors, Senior citizen*  **Geriatrics**  **Geriatric nursing**  Geriatric*  Frail*  Advanced age |
| 2 | End of Life Care | **Terminal Care exp (includes Hospice Care)**  Terminal care*  **Terminally ill**  Terminal* ill*  Terminal disease*  Life limiting illness*  **Hospices**  Hospice*  End of life  Dying  Place of care  Place of death  **Palliative Care**  Palliative |
| 3 | Preferences | **Patient Preference**  Preference*  Wish*  **Attitude to Death**  Attitude to* Death, Death Attitude*  **Decision making**  **Choice behaviour**  Priorities  **Advance Care Planning exp (Includes Advance Directive and Living Will)**  Advance* care plan*  Advance* Directive*  Living will*  **Goals**  Goal* of care, Goal* adj4 Patient* |

Embase

**Subject headings exp= Explode** Truncation*

|  | Category and definition | Search terms (Medline) |
| --- | --- | --- |
| 1 | Older people | **Aged exp (includes Aged hospital patient, aged plant [+NT], frail elderly, institutionalised elderly, very elderly)**  **Geriatrics**  Geriatric*  **Elderly Care exp (includes geriatric care (+NT, inc geriatric nursing), home for the aged, toileting)**  Elder*  Frail*  Older, Oldest Old, Very Old  Seniors, Senior citizen*  Advanced age |
| 2 | End of Life Care | **Terminal Care exp (includes advance care planning, assisted suicide, euthanasia, hospice care)**  Terminal care*  **Terminally ill patient exp (includes hospice patient)**  Terminal* ill*  **Terminal disease**  Terminal disease*  Life limiting illness*  **Hospice**  Hospice*  End of life  **Dying**  Dying  Place of care  Place of death  **Palliative therapy exp (includes cancer palliative therapy)**  Palliative |
| 3 | Preferences | **Patient Preference**  Preference*  Wish*  **Attitude to Death**  Attitude to* Death, Death Attitude*  **Decision making**  **Family decision making**  **Patient decision making**  **Shared decision making**  Choice* adj4 patient*  Priorities  **Advance care planning**  Advance* care plan*  **Living will**  Living will*  Advance* directive*  Goal* of care, Goal* adj4 patient* |

PsychINFO

|  | Category and definition | Search terms (Medline) |
| --- | --- | --- |
| 1 | Older people | **Geriatric patients**  **Geriatrics**  Geriatric*  Elder*  Frail*  Older, Oldest Old, Very Old  Seniors, Senior citizen*  Advanced age |
| 2 | End of Life Care | Terminal care*  **Terminally ill patients**  Terminal* ill*  Terminal disease*  Life limiting illness*  **Hospice**  Hospice*  End of life  **“Death and dying”**  Dying  Place of care  Place of death  **Palliative Care exp**  Palliative |
| 3 | Preferences | **Preferences**  Preference*  Wish*  **Death attitudes**  Death attitude*, Attitude to* Death  **Decision making**  **Choice behaviour exp**  Priorities  **Advance Directives**  Advance* Directive*  Advance* Care Plan*  Living Will*  **Goals**  Goal* of care, Goal* adj4 patient* |

Web of Science

|  | Category and definition | Search terms (Medline) |
| --- | --- | --- |
| 1 | Older people | Elder* OR Older OR “Oldest Old” OR “Very Old” OR Frail* OR Geriatric* OR Seniors OR “Senior citizen*” OR “Advanced age” |
| 2 | End of Life Care | “Terminal care*” OR “Terminal* ill*” OR “Terminal disease*” OR “Life limiting illness*” OR Hospice* OR Palliative OR “End of life” OR Dying OR “Place of care” OR “Place of death” |
| 3 | Preferences | Preference* OR Wish* OR “Attitude to* Death” OR “Death attitude*” OR Decision* OR (choice* NEAR/4 patient*) OR “Advance* Care Plan*” OR “Advance* Directive*” OR “Living Will*” OR "Goal* of care" OR (goal* NEAR/4 patient*) OR Priorities |

Supplementary Material Table 3: Data charting table

| **Study details** | | | | | | | **Aim** |
| --- | --- | --- | --- | --- | --- | --- | --- |
| Authors | Year of publication | Year(s) data collected | Title | Type of reference | Country | Recruitment Context | Aim of study |

| **Study population** | | | | | |
| --- | --- | --- | --- | --- | --- |
| Study population | Study recruitment age | Study population age | Sample size | Participants’ gender | Participants’  ethnicity |

| **Methods** | | | | | | | | **Other** | **Notes** |
| --- | --- | --- | --- | --- | --- | --- | --- | --- | --- |
| Methodology | Method of data collection | Type of data collected | Preference measurement | Scenario | Preferences elicited from (eg patient, carer) | Follow up time | Analysis of preference change/stability | Source of funding |  |

Additional sheets collected detail on:

1. Treatment preferences
2. Hypothetical scenarios
3. Factors which were investigated for correlation with preference change or stability

Supplementary Material Table 4: Key characteristics and results of included references

CHF: Congestive Heart Failure COPD: Chronic Obstructive Pulmonary Disease ED: Emergency Department QoL vs LoL: Judgements about Quality of Life vs Length of Life PPD: Preferred Place of Death RCT: Randomised controlled trial

| **Study** | **Reference** | **Country** | **Recruitment Context** | **Study population** | **Recruitment age** | **Methodology** | **Method of data collection** | **Type of preference** | **Scenario** | **Preferences elicited from** | **Funding** |
| --- | --- | --- | --- | --- | --- | --- | --- | --- | --- | --- | --- |
| 1 | Albert et al 2016^63^  Journal article | USA | Primary care | Older adults participating in a community based cohort study | >=85. | Longitudinal observational quantitative | Structured interview | Treatment preferences | Real-life situation | Participant or Proxy | National Institute on Aging contracts, NIA grant and National Institute of Nursing Research grant. Supported in part by the Intramural Research Program of the NIH, National Institute on Aging. |
|  | Albert et al 2017^64^  Journal article | USA | Primary care | Older adults participating in a community based cohort study | >=85. | Longitudinal observational quantitative | Structured interview | Treatment preferences | Real-life situation | Participant or Proxy | National Institute of Ageing. NIH grants or contracts that support data collection and analysis of the Health ABC. |
| 2 | Auriemma et al 2014^11^  Journal article | Spain, USA, Israel | Systematic review: no limitation on context | Community dwelling older adults in 3 studies | Studies recruiting 'community dwelling older adults' specifically | Systematic review | Database literature searches | N/A | N/A | N/A | Grant from the Doris Duke Charitable Foundation to Perelman School of Medicine at the University of Pennsylvania. Grant from the Otto Haas Charitable Trust. |
| 3 | Barrio-Cantalejo et al 2013^65^  Journal article | Spain | Primary care | Older adults participating in a community based cohort study | >=65 | Longitudinal observational quantitative | Structured interview | Treatment preferences | Hypothetical scenarios | Participant | The Andalusia Health Ministry |
| 4 | Berger et al 1998^66^  Journal article | USA | Primary care | Older nursing home residents | >50 | Longitudinal observational quantitative | Structured interview | Treatment preferences | Real-life situation & Hypothetical scenarios | Participant | Grant from Fan Fox and Leslie R. Samuels Foundation, New York |
| 5 | Blank et al 2001a^67^  Journal article | USA | Secondary care (inpatients) | Older medically hospitalized patients | >60 | Longitudinal observational quantitative | Structured interview | Treatment preferences & PAS, Euthanasia or "wish to die" | Real-life situation & Hypothetical scenarios | Participant | Hartford Hospital Research |
|  | Blank et al 2001b^68^  Journal article | USA | Secondary care (inpatients) | Older medically hospitalized patients | >60 | Longitudinal observational quantitative | Structured interview | PAS, Euthanasia or "wish to die" | Real-life situation & Hypothetical scenarios | Participant | Hartford Hospital Research |
| 6 | Bolt et al 2016^69^  Journal article | Netherlands | Primary care | Older adults participating in two cohort studies. | >=55 | Longitudinal observational quantitative | Structured questionnaire | PAS, Euthanasia or "wish to die" | Real-life situation | Participant and Proxy | The Netherlands Ministry of Health, Welfare and Sport. Grant from Right to Die-NL and The Pieter van Foreest Foundation. Career award from the Netherlands Organization for Scientific Research. |
| 7 | Bosshard et al 2003^70^  Journal article | Switzerland | Primary Care | Nursing home residents | Not stated (nursing home residents) | Longitudinal observational quantitative | Structured interview | Treatment preferences | Hypothetical scenarios | Participant | Not stated |
| 8 | Briggs et al 2021^71^  Journal article | Ireland | Primary Care | Older adults participating in a community based cohort study | >=50 | Longitudinal observational quantitative | Structured interview | PAS, Euthanasia or "wish to die" | Real-life situation | Participant | Irish Government, the Atlantic Philanthropies and Irish Life plc |
| 9 | Bruce-Jones et al 1996^72^  Journal article | UK | Secondary Care (inpatients) | Older patients admitted acutely to medical geriatric units | Not stated (geriatric inpatients) | Longitudinal observational quantitative | Structured interview | Treatment preferences | Real-life situation | Participant | Not stated |
| 10 | Brunner-La Rocca 2012^23^  Journal article | Switzerland, Germany | Secondary Care (outpatients) | Older adults with congestive heart failure | >=60 | Longitudinal interventional quantitative | Structured interview | Treatment preferences, QoL vs LoL | Real-life situation | Participant | Horten Research Foundation and by smaller unrestricted grants from AstraZeneca Pharma, Novartis Pharma, Menarini Pharma, Pfizer Pharma, Servier, Roche Diagnostics, Roche Pharma, and Merck Pharma. |
| 11 | Carmel & Mutran 1999^73^  Journal article | Israel | Primary care | Older Israeli Jews | >=70 | Longitudinal observational quantitative | Structured interview | Treatment preferences, QoL vs LoL | Hypothetical scenarios | Participant | Grant from the US-Israel Binational Science Foundation |
| 12 | Danis et al 1994^74^  Journal article | USA | Primary care | Older adults participating in a community based cohort study | >=65 | Longitudinal observational quantitative | Structured interview | Treatment preferences | Hypothetical scenarios | Participant | University of North Carolina at Chapel Hill, in collaboration with the Cecil G. Sheps Center for Health Services Research. |
| 13 | Ditto et al 2003^75^  Journal article | USA | Primary care | Older adults participating in a community based cohort study | >=65 | Longitudinal observational quantitative  (recruiting from a RCT) | Structured interview | Treatment preferences | Real-life situation & Hypothetical scenarios | Participant | Agency for Healthcare Research and Quality, the Applied Psychology Center at Kent State University and the Summa Health System Foundation. |
|  | Ditto et al 2006^76^  Journal article | USA | Primary care | Older adults who were hospitalised during a cohort study. | >=65 | Longitudinal observational quantitative (recruiting from a RCT) | Structured interview | Treatment preferences | Hypothetical scenarios | Participant | Agency for Healthcare Research and Quality, the Applied Psychology Center at Kent State University and the Summa Health System Foundation. MREP early career award. |
|  | Gready 1999^77^  Dissertation/Thesis | USA | Primary care | Older adults participating in a community based cohort study | >=65 | Longitudinal observational quantitative (recruiting from a RCT) | Structured interview | Treatment preferences | Real-life situation & Hypothetical scenarios | Participant | Not stated |
|  | Gready et al 2000^78^  Journal article | USA | Primary care | Older adults participating in a community based cohort study | >=65 | Longitudinal observational quantitative (recruiting from a RCT) | Structured interview | Treatment preferences | Real-life situation & Hypothetical scenarios | Participant | Agency for Healthcare Research and Quality, the Applied Psychology Center at Kent State University, and the Summa Health System Foundation |
|  | Houts et al 2002^79^  Journal article | USA | Primary care | Older adults participating in a community based cohort study | >=65 | Longitudinal observational quantitative (recruiting from a RCT) | Structured interview | Treatment preferences | Real-life situation & Hypothetical scenarios | Participant | Agency for Health Care Policy and Research, Kent State University and the Summa Health System Foundation. A National Institute of Child Health and Human Development postdoctoral fellowship at the Center for Developmental Science, University of North Carolina at Chapel Hill |
|  | Sharman et al 2008^62^  Journal article | USA | Primary care | Older adults participating in a community based cohort study | >=65. | Longitudinal observational quantitative (recruiting from a RCT) | Structured interview | Treatment preferences | Hypothetical scenarios | Participant | Not stated |
| 14 | Eggar et al 2002^80^  Journal article | UK | Secondary care (outpatients and inpatients) | Day patients and inpatients of a geriatric psychiatric unit | >=65 | Longitudinal observational quantitative | Structured interview | Treatment preferences | Real-life situation | Participant | Not stated |
| 15 | Eneslatt et al 2021^24^  Journal article | Sweden | Primary care | Older community-dwelling adults without imminent end-of-life care needs | Not stated (Older adults) | Longitudinal qualitative | In-depth semi-structured interview supported by an EcoMap and DoBra (GoWish) cards | Goals of care | Real-life situation | Participant | Swedish Research Council for Health, Welfare and Working Life; Doctoral School for Health Care Sciences at Karolinska Institutet; Vårdal Foundation; Göteborg Center for Person-Centered Care; Strategic Research Area Health Care Sciences at Karolinska Institutet and Umeå University; The Dementia Foundation; Ulrika Cronés Foundation; and Sahlgrensringen Foundation. An endowment from Investor AB. |
| 16 | Etkind et al 2020^29^  Journal article | UK | Primary and Secondary care | Older adults with frailty and recent acute illness | >=65 | Longitudinal mixed method | Structured interview (all participants). In-depth semi-structured interview (17 participants). | Goals of care | Real-life situation | Participant | Cicely Saunders International & the Atlantic Philanthropies |
| 17 | Fried & Bradley 2003^81^  Journal article | USA | Secondary care (outpatients) | Community dwelling older adults with advanced CHF, COPD or cancer. | >=60 | Cross-sectional qualitative | In-depth, semistructured interview and focus group | Treatment preferences | Real-life situation | Participant | Paul Beeson Physician Faculty Scholars Award and a Veterans Affairs HSR&D Career Development Award |
| 18 | Fried & O'Leary 2008^82^  Journal article | USA | Secondary care (outpatients and inpatients) | Caregivers of community dwelling patients who had died with advanced CHF, COPD or cancer. | >=60 (Patients) | Cross-sectional qualitative | In-depth, semistructured interview | Treatment preferences | Real-life situation | Proxy | VA Health Services Research & Development (HSR&D) and the National Institute on Aging and a Paul Beeson Physician Faculty Scholars Award. |
| 19 | Fried et al 2006^83^  Journal article | USA | Secondary care (outpatients and inpatients) | Community dwelling older adults with advanced CHF, COPD or cancer. | >=60 | Longitudinal observational quantitative | Structured interview | QoL vs LoL | Hypothetical scenarios | Participant | VA HSR&D, the National Institute on Aging, the Claude D. Pepper Older Americans Independence Center at Yale University and by a Paul Beeson Physician Faculty Scholars Award. |
|  | Fried et al 2007a^84^  Journal article | USA | Secondary care (outpatients and inpatients) | Community dwelling older adults with advanced CHF, COPD or cancer. | >=60 | Longitudinal observational quantitative | Structured interview | Treatment preferences | Hypothetical scenarios | Participant | VA HSR&D, the National Institute on Aging, the Claude D. Pepper Older Americans Independence Center at Yale, a Paul Beeson Physician Faculty Scholars Award and the National Institute of Arthritis and Musculoskeletal and Skin Diseases. |
|  | Fried et al 2007b^85^  Journal article | USA | Secondary care (outpatients and inpatients) | Community dwelling older adults with advanced CHF, COPD or cancer. | >=60 | Longitudinal observational quantitative | Structured interview | Treatment preferences | Hypothetical scenarios | Participant | Grants from VA HSR&D, the National Institute on Aging, the Claude D. Pepper Older Americans Independence Center at Yale and a Paul Beeson Physician Faculty Scholars Award. |
|  | Casarett et al 2006^86^  Journal article | USA | Secondary care (outpatients and inpatients) | Community dwelling older adults with advanced CHF, COPD or cancer. | >=60 | Longitudinal observational quantitative | Structured interview | Treatment preferences | Real-life situation & Hypothetical scenarios | Participant | VA HSR&D and the National Institute on Aging. Advanced Research Career Development Award from the Department of Veterans Affairs and a Paul Beeson Physician Faculty Scholars Award. |
| 20 | Gallo et al 2019^87^  Journal article | USA | Primary care | "Older physicians" in a cohort study | >60 | Longitudinal observational quantitative | Structured questionnaire | Treatment preferences | Hypothetical scenarios | Participant | National Institute of Nursing Research |
|  | Wittink et al 2008^88^  Journal article | USA | Primary care | "Older physicians" in a cohort study | >60 | Longitudinal observational quantitative | Structured questionnaire | Treatment preferences | Hypothetical scenarios | Participant | NIMH Mentored Patient-Oriented Research Career Development Award. Grants from National Institutes of Health. |
| 21 | Ganzini et al 1994^89^  Journal article | USA | Secondary Care (inpatients) | Older psychiatric inpatients diagnosed with depression | >60 | Longitudinal observational quantitative | Structured interview | Treatment preferences | Real-life situation & Hypothetical scenarios | Participant | Grants from the Medical Research Foundation of Oregon and the VA Merit Review Program. |
| 22 | Hanson et al 2023^90^  Journal article | Denmark | Secondary care (inpatients) | Patients admitted to hospital via the ED with a medical condition | >=65 | Longitudinal observational quantitative | Structured questionnaire and structured interview | Treatment preferences | Real-life situation | Participant | Region of Southern Denmark [16/42004], the Health Foundation [17-B-0072], and the Karola Jorgensens Foundation |
| 23 | Hickman et al 2021^91^  Journal article | USA | Primary care | Nursing facility residents and surrogate decision makers | >=65 (Residents) | Cross-sectional qualitative | Semi-structured interviews | Treatment preferences | Real-life situation | Participant or Proxy | National Institute of Nursing Research |
| 24 | Hooper et al 1997^92^  Journal article | Australia | Secondary care (outpatient and inpatient) | Older patients being treated for major depression | Not stated (psycho-geriatric patients) | Longitudinal observational quantitative | Structured questionnaire | PAS, Euthanasia or "wish to die" | Real-life situation & Hypothetical scenarios | Participant | Not stated |
| 25 | Kawaguchi et al 2022^93^  Journal article | Japan | Primary care | Older non institutionalised adults | >65 | Longitudinal observational quantitative | Structured questionnaire | PPD | Real-life situation | Participant | Japan Society for the Promotion of Science, Health Labour Sciences Research Grants, Ministry of Health, Labour and Welfare, Japan Agency for Medical Research and development, National Center for Geriatrics and Gerontology, Science and Technology, Foundation For Aging And Health, Innovative Research Program on Suicide Counter measures, Sasakawa Sports Foundation, Health Promotion & Fitness Foundation, Chiba Foundation for Health Promotion & Disease Prevention, 8020 Promotion Foundation, Meiji Yasuda Life Foundation of Health and Welfare. |
| 26 | Lee et al 1994^94^  Journal article | USA | Secondary Care (inpatients) | Depressed and non-depressed medical inpatients | >65 | Longitudinal observational quantitative | Structured questionnaire | Treatment preferences | Real-life situation & Hypothetical scenarios | Participant | The Hearst Foundation and the Medical Research Foundation of Oregon |
| 27 | Lockhart et al 2001^95^  Journal article | USA | Primary care | Older adults | >=65. | Longitudinal observational quantitative | Structured interview | QoL vs LoL | Hypothetical scenarios | Participant | Agency for Health Care Policy and Research, the Applied Psychology Center at Kent State University, and the Summa Health System Foundation. |
| 28 | Martin & Roberto 2006^27^  Journal article | USA | Primary care | Older adults | Not stated (older adults) | Longitudinal mixed method | Structured interview and Semi-structured interview | Treatment preferences | Real-life situation & Hypothetical scenarios | Participant | Not stated |
| 29 | McCaughan 2019^96^  Journal article | UK | Secondary care (outpatients) | Bereaved relatives of older adults who had died from a haematological malignancy | Not stated | Cross-sectional qualitative | In-depth, semistructured interviews | Preferred place of death | Real-life situation | Proxy | Marie Curie Research Grants Scheme. The Haematological Malignancy Research Network was funded by Bloodwise. |
| 30 | McParland 2003^97^  Journal article | USA | Primary care | Nursing home residents | >=65. | Longitudinal observational quantitative | Structured interview | Treatment preferences | Real-life situation | Participant | American Federation for Aging Research/John A. Hartford Foundation Fellowship |
| 31 | Ross & Austin 2015^25^  Journal article | UK | Secondary care (outpatients) | Patients with end stage heart failure | Not specified | Longitudinal qualitative | Semi-structured interview | Spiritual preferences | Real-life situation | Participant and Proxy | Nevill Hall Thrombosis & General Research Fund, the Cardiology Charitable Fund and the University of Glamorgan (now University of South Wales). |
| 32 | Skolarus et al 2022^98^  Journal article | USA | Primary care | Older adults participating in a community based cohort study | >=65 | Longitudinal observational quantitative | Structured interview | Treatment preferences | Hypothetical scenarios | Participant | National Institutes of Health and National Institute on Ageing |
| 33 | Somogyi-Zalud et al 2000^99^  Journal article | USA | Secondary Care (inpatients) | Hospitalised patients aged 80 and over- patients who died during the cohort study | >=80 | Longitudinal observational quantitative | Structured interview | Treatment preferences and QoL vs LoL | Real-life situation & Hypothetical scenarios | Participant and Proxy | Grants from the Robert Wood Johnson Foundation. Career Development Award from the National Institute on Aging. |
|  | Tsevat et al 1998^100^  Journal article | USA | Secondary Care (inpatients) | Hospitalised patients aged 80 and over | >=80 | Longitudinal observational quantitative | Structured interview | QoL vs LoL | Real-life situation | Participant | Grant from the Robert Wood Johnson Foundation. |
| 34 | Stegmann et al 2020^30^  Journal article | Netherlands | Secondary care (outpatients) | Older patients with non-curable cancer. | >=60 | Longitudinal mixed method (recruiting from a RCT) | Outcome Prioritisation Tool (Structured questionnaire) and Medical record review | Goals of care | Real-life situation | Participant. Medical record. | Grant from the Dutch Cancer Society Fund |
| 35 | van Doorne et al 2021^101^  Journal article | Netherlands | Secondary Care (inpatients) | Older patients with palliative care needs who were admitted acutely to hospital. | >=65 | Longitudinal observational quantitative (recruiting from a RCT) | Structured interview | PPD | Real-life situation | Participant | ZonMw- The Netherlands Organisation for Health Research and Development |
| 36 | van Wijngaarden et al 2021^26^  Journal article | Netherlands | Primary care | Older adults who were not terminally ill but self-identified as having a wish to die. | >=70 | Longitudinal qualitative | Phenomenological interview and textual data from participants' emails and obituaries | PAS, Euthanasia or "wish to die" | Real-life situation | Participant and Proxy | ZonMw- The Netherlands Organization for Health Research and Development |
| 37 | Waldrop & Meeker 2014^102^  Journal article | USA | Primary and Secondary care (community hospice) | Community hospice patients and their caregivers | >=65 | Cross-sectional qualitative | In-depth semi-structured interview | Goals of care, PPD and Decision making | Real-life situation | Participant and Proxy | Grant from Buffalo Center for Social Research |
| 38 | Walker et al 2018^103^  Journal article | USA | Primary care | Older patients with >=2 chronic health conditions | >=60 | Longitudinal observational quantitative | Medical record review | Treatment preferences | Real-life situation | Medical record- where there was evidence of patient involvement in decisions. | Funded by U.S. Department of Veterans Affairs |
| 39 | Watson et al 1997^28^  Journal article | New Zealand | Secondary care (inpatients) | Older inpatients without terminal illness | Not stated (Elderly inpatients) | Longitudinal mixed method | Semi-structured interview | Treatment preferences and decision making preferences | Real-life situation | Participant | Funded by Canterbury Health Care for the Elderly Education Trust |
| 40 | Yamaguchi et al 2017^104^  Letter to the Editor | Japan | Secondary care (outpatients) | Geriatric outpatients | Not stated (geriatric outpatients) | Longitudinal observational quantitative | Structured questionnaire | Treatment preferences | Hypothetical scenarios | Participant | Not stated. |
